# Seasonal variability of faecal indicator bacteria numbers and die-off rates in the Red River basin, North Viet Nam

**DOI:** 10.1038/srep21644

**Published:** 2016-02-12

**Authors:** Huong Thi Mai Nguyen, Quynh Thi Phuong Le, J. Garnier, J.-L. Janeau, E. Rochelle-Newall

**Affiliations:** 1 Institute of Natural Product Chemistry, Viet Nam Academy of Science and Technology, 18 Hoang Quoc Viet Road, Cau Giay, Hanoi, Viet Nam; 2 Institut de Recherche pour le Développement (IRD), iEES -Paris, UMR 242, Centre IRD Nord, 32 avenue Henri Varagant, Bondy, F-93143, France; 3 UMR METIS, Université de Pierre et Marie Curie, 4 Place Jussieu, 75005, Paris, France; 4 Institut de Recherche pour le Développement (IRD), iEES-Paris UMR 242, c/o Soils and Fertilizers Research Institute (SFRI), Hanoi, Viet Nam

## Abstract

The Red River is the second largest river in Viet Nam and constitutes the main water source for a large percentage of the population of North Viet Nam. Here we present the results of an annual survey of *Escherichia coli* (EC) and Total Coliforms (TC) in the Red River basin, North Viet Nam. The objective of this work was to obtain information on faecal indicator bacteria (FIB) numbers over an annual cycle and, secondly, to determine the die-off rates of these bacterial indicators. Monthly observations at 10 stations from July 2013–June 2014 showed that TC and EC reached as high as 39100 cfu (colony forming units) 100 ml^−1^ and 15300 colonies 100 ml^−1^, respectively. We observed a significant seasonal difference for TC (p < 0.05) with numbers being higher during the wet season. In contrast, no significant seasonal difference was found for EC. The FIB die-off rates ranged from 0.01 d^−1^ to a maximum of 1.13 d^−1^ for EC and from 0.17 d^−1^ to 1.33 d^−1^ for TC. Die-off rates were significantly higher for free bacteria than for total (free + particle attached) bacteria, suggesting that particle attachment provided a certain level of protection to FIB in this system.

Biological contamination of aquatic systems by water borne pathogens from untreated wastewater and agricultural effluent is a globally important water quality problem^1^. However, it is particularly problematic in tropical regions where a large proportion of the developing world is located. On a global scale, it is estimated that 88% of diarrheal diseases are due to the use of unclean water sources, leading to the deaths of 1.8 million people annually, most of whom are children in developing countries^2^. Indeed, in many developing countries, surface water (e.g. rivers and stagnant ponds) subject to wastewater contamination is often used for domestic purposes as access to uncontaminated water is limited^3^. Therefore, considering the high death rates as well as the large economic burden associated with the construction and maintenance of water treatment plants, having an understanding of the spatial distribution and temporal variability of the microbial pathogens responsible for these diseases is essential. Furthermore, understanding the factors that control their distribution is a prerequisite for reducing the human health risks associated with the use of unclean water. This is particularly important in tropical areas where there is a paucity of data, where population growth is high, and where populations are the most exposed to these contaminants^1^,^3^.

Rivers are a major source of fresh water for irrigation, industry and domestic water requirements. However, many tropical rivers have been adversely affected by human activities, such as industrialization, urbanization and agricultural intensification^4^,^5^. Although the chemical contamination of water bodies has been documented in many tropical systems^6^,^7^, the extent of biological contamination from untreated wastewater and animal husbandry is often unknown. This is despite the fact that detailed knowledge on the range and origin of microbial pollution is required for watershed management in order to provide safe water for human demands.

The Red River is the second largest river in Viet Nam, after the Mekong River, and one of the five largest rivers in East Asia^8^. Over 24 million inhabitants live in the Red River basin, including over 17 million people in its delta. This area is also characterized by several large industrial zones and by a large number of craft villages that are considered hotspots of biological and chemical contamination^9^. The Red River Delta (RRD) is the second most important rice-producing area in Viet Nam and accounts for 20% of the national production. It is also the main freshwater source for the surrounding areas as well as being the major outlet for wastewater^10^,^11^. According to the Viet Nam Environment Administration Report 2012, the urban area of the RRD concentrates 24% of the national production of domestic wastewater. It also receives the second largest proportion of industrial wastewater in the country after that of the South East region around Ho Chi Minh City. Despite the high proportions of wastewater that are released into the Red River on a daily basis, little information exists in the published literature on microbial or faecal contamination levels in this semi-tropical region.

Faecal indicator bacteria (FIB) are used to monitor faecal contamination levels and hence the possibility of pathogens of faecal origin in soils and water in both tropical and temperate systems^12^,^13^. FIB is a generic term for a range of bacteria that inhabit the gastrointestinal tract of homoeothermic animals. This group includes *Escherichia coli*, *Salmonella spp.*, *Enterococcus spp*., and the coliforms. We hypothesized that FIB numbers would increase along the river length as a consequence of the increasing industrialization and urbanization in the downstream sections. Here we present the results of an annual survey of FIB at ten stations along the Red River, North Viet Nam. The objective of this work was to obtain information on FIB concentrations over one annual cycle and to identify the environmental factors controlling FIB numbers and to determine their die-off rates.

## Materials and Methods

### Study site

The Red River basin has an area of about 156 451 km^2^ of which 51.2% is in Viet Nam, 47.9% in China and 0.9% in Laos^14^. The basin is subject to a semi-tropical climate with two clear seasons. The wet season persists from May to October during which 80–90% of the total annual rainfall of 1900 mm occurs^15^. The cooler, dry season persists from November to April. Mean monthly temperatures are lowest in January, with June-August being generally the hottest. In general, temperature is relatively uniform across the basin and the mean relative humidity is greater than 80%^16^. Concomitant with the highest rainfall, discharge and suspended load peak during August in the middle of the wet season^14^.

Samples were collected monthly from July 2013 to June 2014 at 10 stations (total of 120 samples) in the Red River Basin. The sample sites are located on different river branches (distributaries) of the Red River system and include, from upstream to downstream, Yen Bai (Thao River), Hoa Binh (Da River), Vu Quang (Lo River), Gian Khau (Day river), Truc Phuong (Ninh Co River), Quyet Chien (Tra Ly River), Nam Dinh (Dao River) and Son Tay, Ha Noi and Ba Lat on the main axe of the Red River (Table 1).

### Sample collection

At each sample site, 1500 ml of river water was collected with a plastic bottle that had been acid washed (10% HCl), rinsed copiously with distilled water and dried. The samples were stored in a cooler and returned to the laboratory for processing. The sample was used to measure dissolved oxygen (DO), pH, conductivity, temperature, salinity, total suspended solids (TSS), total phosphorus (TP), dissolved inorganic phosphate (PO_4_), ammonia-nitrogen (NH_3_-N) and free and attached FIB.

### Die-off rates

At four stations, a second series of samples was collected in the same way for the determination of FIB die-off rate over time. These stations were (1) Yen Bai, located in the upstream main branch of the Red River known as the Thao River; (2) Ha Noi, after the confluence of three major upstream tributaries of the Da, Thao and Lo rivers; (3) Gian Khau, a peri-urban river system located in the Red River Delta and, (4) Truc Phuong, located in the downstream Red River on the Ninh Co River. These four stations were chosen to give a good representation of the land uses and population densities in the basin and to provide a good geographical separation over the area. For each station, duplicate 750 ml samples were incubated for five days in glass bottles in the dark and at the *in situ* temperature of the Hanoi station (17–29 °C). This temperature was selected for two reasons. The first being that only one incubator was available for the experiment and the second being that temperature at the Hanoi station was close to the average of the temperatures at the other stations. For the estimation of die-off rates, samples were collected from the incubations every day during 5 days (T0, T1, T2, T3, T4, T5) to determine the decrease in FIB numbers for both total and free bacteria using the method described below.

### Analytical methods

Temperature, dissolved oxygen (DO), pH, and total suspended solids (TSS) were measured using a water quality probe WQC-22 A (TOA, Japan) and conductivity (Cond) was determined using a conductivity meter (Hach, USA) immediately upon sampling. Nutrients (N, P, Si) were spectrophotometrically determined on a Drell 2800 (HACH, USA) in the laboratory according to APHA^17^ methods.

FIB abundance (free and attached) was measured by a direct count method using 3 M Petrifilm™ E.coli/Coliform Count Plate (Petrifilm EC plate), which contain Violet Red Bile (VRB) nutrients. *E.coli* (EC) produces beta-glucuronidase, which produces a blue precipitate and Total coliform (TC) colonies growing on the Petrifilm EC plate produce acid and the colonies are denoted by dark red points. This method has been validated by the APHA and is a technique commonly used for coliform and EC counts^18^,^19^.

For the total counts (free + attached bacteria; EC_tot_ and TC_tot_), 1 ml was removed from each sample (or incubation) after shaking to ensure an even distribution of bacteria, the sample was then aseptically delivered to the center of a Petrifilm EC plate. The water sample was then left to stand for 1 h and a second 1 ml aliquot was inoculated onto a second Petrifilm EC plate to estimate the number of free EC (EC_free_ or TC_free_; i.e. non-sedimented). The Petrifilm EC plates were then incubated at 37 °C for 24 hours, using a Fukusima incubator (Japan). The number of colonies (EC and TC) was determined using a Colony Counter CL-560 (Sibata, Japan). To facilitate the comparison of our data with that of previously published data and with water quality limits, we express our data as the number of colonies per 100 ml of sample.

The number of attached EC or TC (EC_att_ or TC_att_) is determined from the difference between EC_tot_ (or TC_tot_) and EC_free_ (or TC_free_) as:


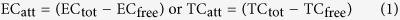


and the percent of attached EC or TC (%EC_att_ or %TC_att_) calculated as:


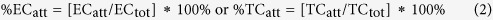


The die-off rates of TC and EC were estimated by fitting an exponential equation to bacterial abundances measured over time. The equations were expressed as first order decay in the general form of:


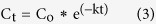


where C_t_ = is the number of EC or TC at elapsed time t, C_o_ is the initial number of EC or TC per ml, k is the decay constant in day^−1^ and t is the elapsed time in days. As in several incubations complete die-off was observed by day 4, k is calculated for the first 4 days for all incubations to ensure comparability between dates and stations. The k value was determined for both the free and total fractions (e.g. EC_free_, TC_free_, EC_tot_, and TC_tot_).

All statistical analyses were performed with XLSTAT (v. 2014). Pearson’s correlation was used to test the relationships between environmental variables and FIB. Wilcoxon’s non-parametric test was used to test for significant differences between variables and the Kruskal-Wallace test was used to test differences between stations and season as the data were non-normally distributed even after normalization. When statistical relationships concerning FIB number were tested, log(EC) or log(TC) was used. When a significant difference was observed, an *a posteriori* Dunn’s all-pairwise test was used and significance is determined as p < 0.05.

## Results

### Physico-chemical variables

**Figure 1:**
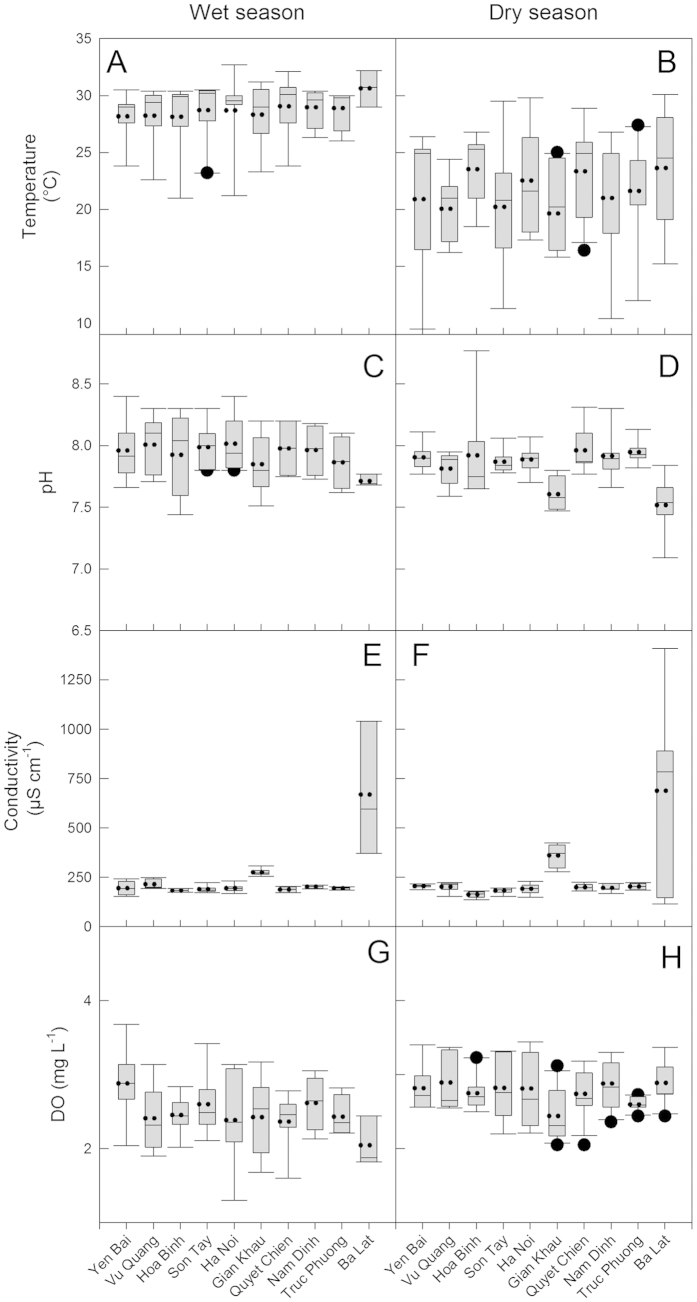
Box plots of temperature, pH, conductivity and DO concentrations for each station for the wet (May to October) and the dry (November to April) seasons for the study period (July 2013 to June 2014). Left side panels are for the wet season and the right hand side panels are for the dry season. Panels (**A,B**): Temperature, panels (**C,D**): pH, panels (**E,F**): conductivity and panels (**G,H**): DO. Mean (dotted line), median (solid line) and whiskers (error bars) above and below the box indicating the 90th and 10th percentiles are shown.

**Figure 2:**
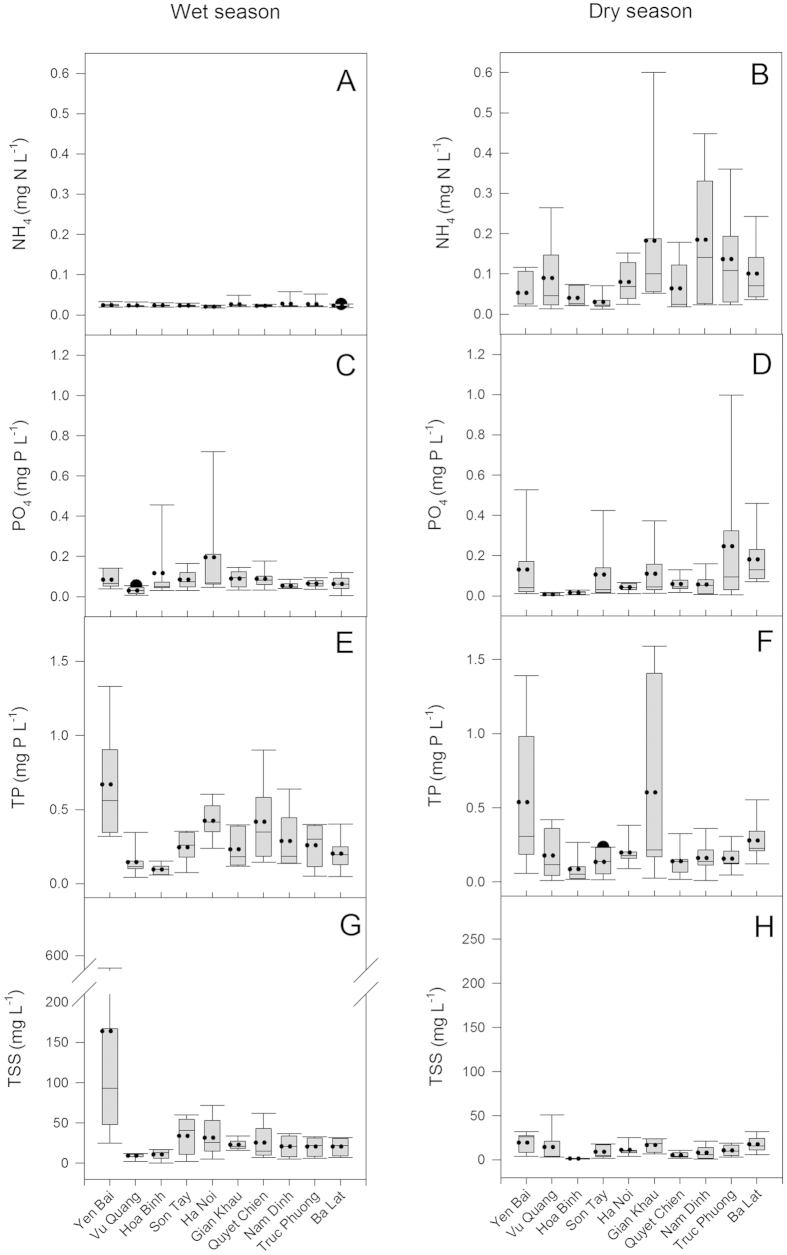
Box plots of NH_4_, PO_4_, TP and TSS concentrations for each station for the wet (May to October) and the dry (November to April) seasons for the study period (July 2013 to June 2014). Left side panels are for the wet season and the right hand side panels are for the dry season. Panels (**A,B**): NH_4_, panels (**C,D**): PO_4_, panels (**E,F**): TP and panels (**G,H**): TSS. Mean (dotted line), median (solid line) and whiskers (error bars) above and below the box indicating the 90th and 10th percentiles are shown.

The variability of the physico-chemical parameters observed in the ten stations for the wet and dry seasons are presented in Figs 1 and 2. Temperature varied between 9.5 and 35° C and was significantly higher during the wet season (p < 0.0001). Temperature was generally highest at Ba Lat and lowest at Gian Khau.

The pH ranged from ranged 7.1 to 8.8, with the lowest values observed at the downstream Ba Lat station and the highest at upstream Hoa Binh station, however, no seasonal differences were observed. Salinity was almost 0 at all stations during the entire year; the only exception was during March at the most downstream station (Ba Lat, located at the river mouth and under tidal influence) where a salinity of 8.5 was observed (data not shown). The highest conductivities were also observed at this station at this time (1410 μScm^−1^). At the other stations, conductivity varied between 136–423 μScm^−1^ (Fig. 1) and had no significant seasonal pattern.

Concentrations of NH_4_, PO_4_ and TP are shown in Fig. 2. Ammonium varied from 0.01 to 0.6 mg N l^−1^ and was significantly higher during the dry season (November – April) at low dilution and in the downstream stations (Gian Khau, Nam Dinh, Truc Phuong and Ba Lat) of the highly populated delta area. Similar to NH_4_, the PO_4_ tended to be higher during the dry season however the difference was not significant. TSS was significantly higher during the wet season, when particle load is higher (p < 0.0001). Overall, TSS concentrations were significantly higher at the upstream Yen Bai station (p < 0.03) than at the other stations. As for TSS, TP was significantly higher during the wet season (p < 0.012). High concentrations of TP were found at Yen Bai and Gian Khau during the dry season (January) when values of up to 1.39 and 1.59 mg PO_4_ l^−1^ were observed.

### FIB abundance: monthly observation

**Figure 3:**
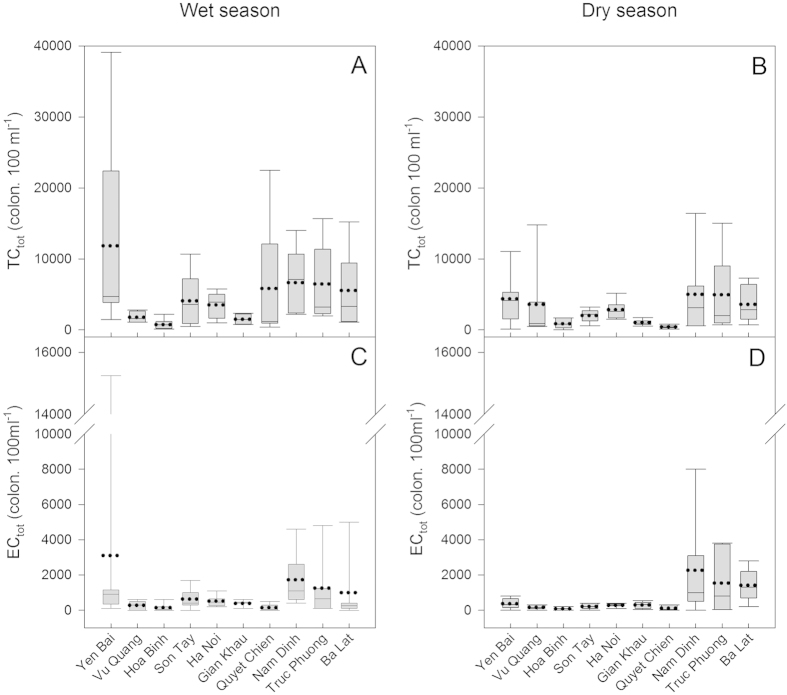
Box plots of the number colonies of TC_tot_ and EC_tot_ for the wet season (left hand side) and dry season (right hand side) for the ten stations. Panels (**A,B**): TC_tot_, panels (**C,D**): EC_tot_. Mean (dotted line), median (solid line) and whiskers (error bars) above and below the box indicating the 90th and 10th percentiles are shown.

The EC_tot_, and TC_tot_ numbers for each season at the ten different stations are shown in Fig. 3 and Table S1 (Supplementary Materials). The number of TC_tot_ varied between 0 colonies 100 ml^−1^ at Hoa Binh in January to over 39100 colonies per 100 ml^−1^ at Yen Bai in September. During the wet season, TC_tot_ at Yen Bai, the most upstream station, was 9783 ± 14431 (mean ± standard deviation) colonies 100 ml^−1^ as compared to 4383 ± 3797 colonies 100 ml^−1^ for this station during the dry season, with no significant difference between these two seasons. For the other upstream tributaries, relatively low mean TC_tot_ numbers were observed during the wet season (1850 ± 715 and 800 ± 789 colonies 100 ml^−1^ for Hoa Binh and Vu Quang, respectively. Mean values in the downstream delta stations were 6066 ± 4506, 5408 ± 5379 and 6050 ± 5469 colonies 100 ml^−1^ for Nam Dinh, Truc Phuong and Ba Lat, respectively. The overall pattern was similar for the dry season, i.e. higher mean values at Yen Bai and in downstream delta stations such as the peri-urban Nam Dinh station where a seasonal average of 5016 ± 5840 colonies 100 ml^−1^ was found. When the data from all of the stations were combined TC_tot_ exhibited a significant seasonal difference with lower numbers during the dry season as compared to the wet season (Fig. 3; p = 0.042).

EC_tot_ numbers were significantly lower than TC_tot_ (p < 0.05) during both seasons at all of the stations. As for TC_tot_, low numbers of EC_tot_ were found in the upstream tributaries during wet season (283 ± 248 and 150 ± 234 colonies 100 ml^−1^ at Vu Quang and Hoa Binh, respectively), and high numbers were observed at Yen Bai (3108 ± 5960 colonies 100 ml^−1^). The more anthropogenically impacted downstream stations of the delta had high values during wet season (e.g. 1733 ± 1632 colonies 100 ml^−1^ at Nam Dinh). During the dry season, a similar pattern was observed with high mean EC_tot_ numbers at Nam Dinh (2266 ± 3007 colonies 100 ml^−1^) and low values in the upstream stations (167 ± 122, 83 ± 75.7 colonies 100 ml^−1^ for Vu Quang and Hoa Binh, respectively). Regarding the dataset as a whole, there was no significant difference between the wet and dry seasons for EC_tot_.

### Particle attached FIB

**Figure 4:**
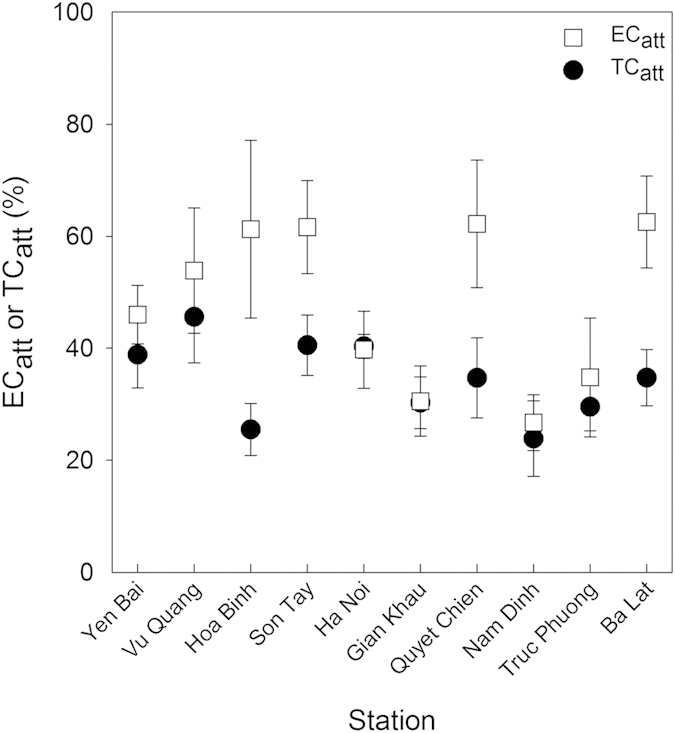
Percentage TC_att_ and EC_att_ for each of the 10 stations. The mean and standard error for each station are given. Filled circles: TC_att_, open squares: EC_att._

The percentage of EC_att_ and TC_att_ varied greatly between station (Fig. 4, Table S1). For example, the percentage of EC_att_ varied between 9.7% and 100% with a mean ( ± se) of 46.5 ± 2.9% (Table S1). The percentage of TC_att_ was also highly variable (2.7–100%) with a mean 34.8 ± 1.8% for the whole data set combined. The number of EC_att_ or TC_att_ was significantly lower than for EC_free_ or TC_free_ when the entire dataset was examined (p < 0.05).

The number of EC_att_ differed significantly between station (p < 0.05) and was highest at Yen Bai (4900 cells 100 ml^−1^) and lowest at Hoa Binh (100 colonies 100 ml^−1^; Table S1), similar to what was observed for the total number (free + attached). As with EC_att_, the number of TC_att_ differed significantly between station. TC_att_ was highest at Yen Bai (13050 colonies 100 ml^−1^) and the lowest at Hoa Binh (0 colonies 100 ml^−1^). The numbers of free and attached TC and EC were positively correlated with each other and had correlation coefficients of 0.73 and 0.81, respectively. This indicates that attached and free TC and EC increased concomitantly even though the actual numbers were different.

### Die-off rates

The die-off rates of EC and TC calculated over 4 days are shown in Table 2. In general, die-off rates for TC_tot_ were significantly higher than for EC_tot_. For example, at Yen Bai in August, the die-off rate for TC_tot_ was 1.33 d^−1^ compared to 0.50 d^−1^ for EC_tot_ and 1.03 d^−1^ and 0.43 d^−1^ TC_tot_ and EC_tot_ respectively, at Truc Phuong during February. It is worth mentioning again the specific behavior of FIB at Yen Bai. Along with the high TC and EC numbers at this station, TC_tot_ die-off rates were higher than at the other three stations examined e.g. 1.33 d^−1^ as compared to 0.70 d^−1^, 1.03 and 0.42 d^−1^ for Hanoi, Truc Phuong and Gian Khau, respectively. Similarly, EC_tot_ die-off rates at Yen Bai were also significantly higher (p < 0.05) than those observed for the three other stations. However, and in contrast to TC_free_, we observed a significant station effect for EC_free_ with the rates found at Yen Bai and Truc Phuong being significantly higher than those found at the two other stations, Hanoi and Gian Khau. Overall, die-off rates were significantly higher for TC_free_ than for TC_tot_ (p < 0.05) when the whole data set was examined. In contrast, the opposite was true for EC_tot_ and EC_free_ (p < 0.05).

### Relationships between FIB and environmental factors

When the data set was regarded either as a whole or by station, EC and TC (free, attached and total) numbers were only positively correlated with TSS and TP (p < 0.05; Table 3). No other significant relationship was found between EC or TC and the measured environmental variables (pH, DO, NH_4_, conductivity, or PO_4_).

As for TC_tot_ and EC_tot_ numbers, the die-off rates of both TC_free_ and EC_free_ and TC_tot_ and EC_tot_ were uncorrelated with temperature, DO, NH_4_ or conductivity (Table 4). However, EC_tot_ die-off rate was significantly correlated with TP and TSS. No significant correlations were observed for TC_tot_ and TC_free_ and TSS.

## Discussion

### Distribution of FIB

Land use affects the abundance and distribution of FIB in both tropical^20^,^21^ and temperate regions^22^,^23^. It is also probably the case in the Red River system. In general, mean FIB numbers (EC, TC) in the upstream stations (e.g. Vu Quang, Hoa Binh, Son Tay) were lower than the downstream stations (Nam Dinh, Truc Phuong and Ba Lat). Of the ten stations investigated, the upstream Hoa Binh station on the Da River had the lowest FIB levels. The Da River sub-basin is primarily forest (74% of total sub-basin area) and population density is sparse (<100 people per km^−2^) and this probably explains the low FIB numbers at this station. In contrast, Yen Bai on the Thao River has the highest and most variable levels of contamination, despite being upstream. The Thao River basin has a relatively high population density (190 people km^−2^) and a large proportion of the total sub-basin area (33%) is used for agriculture and livestock grazing. Livestock are known to be an important source of FIB contamination in streams and rivers^20^ and this along with non-treated human wastewater from the surrounding population may well explain the high and variable FIB numbers observed at this site during both seasons. Furthermore, the Van Yen industrial zone which processes agricultural products is located in the upstream Thao River and this is probably also a source of FIB. Indeed, the high and variable FIB numbers observed during the wet season also suggest that FIB originated mostly from diffuse runoff sources.

The higher FIB abundances observed in the downstream stations (Nam Dinh, Ba Lat and Truc Phuong) are also probably related to the surrounding land use. These three sites are in peri-urban areas of the delta with high population densities (1200 people km^−2^ compared to 100–150 people km^−2^ in the upstream basin^14^), agriculture and industry all of which release untreated wastewater into the system. Although the Hanoi station is located in the city and the FIB numbers are relatively low (Fig. 3), they are still above the acceptable limits for individual, non-commercial water supplies in Viet Nam (20 EC colonies 100 ml^−1^ and 150 TC colonies 100 ml^−1^)^24^. Domestic wastewater from Hanoi city is mostly discharged into small urban rivers as shown by the high ammonium concentrations in the To Lich and Nhue Rivers^10^,^25^,^26^ and other work from the urban rivers in Hanoi city has reported very high TC numbers. For example, TC numbers at Lien Mac, at Phu Van Bridge (Nhue River) and at Thanh Liet Dam (To Lich river) were 7820, 9820 and 148480 MPN 100 ml^−1^, respectively^27^ meaning that the water at these locations is considered as being unfit even for irrigation due to its high FIB load.

### Free and attached FIB

Understanding the relative numbers of attached and free FIB and their differential fate allows us to better estimate the time these indicator organisms remain viable in the environment. In soils and sediments, bacteria tend to be associated with particles as opposed to in the free-state e.g.^28^ whereas in aquatic systems the percentages of particle associated bacteria are highly variable and can range from 10% in clear waters with very low organic particle loads to over 70% in estuaries with high particle loads^29^,^30^. Suter *et al*.^31^ have shown that high proportions of FIB are associated with particles (52.9% ± 20.9% and over 90% in some areas) and that these values were related to turbidity levels.

In our work, free-living bacteria generally predominated (only 36% ± 20% of TC were particle attached and 50% ± 26.9% of EC particle attached). However, the %EC_att_ and %TC_att_ were highly variable (7.8% to 100% and 2.7% to 80% for EC_att_ and TC_att_, respectively). Previous studies from temperate systems have indicated that the attachment to particles by FIB in the aquatic environment is influenced by various factors, including temperature, bacterial genotype, soil particle size, organic matter, pH, ionic strength, dissolved nutrients and turbidity^32^,^33^. We also found significant correlations among TC_att_, EC_att_, TSS and TP. In one of the few articles investigating the factors controlling FIB concentrations in the tropics, Byamukama *et al*.^34^ working in Uganda, also found that FIB were correlated with TSS concentrations as we show here. The Red River system has high turbidity levels^8^,^14^ and this may explain the relatively high %EC_att_ and %TC_att_ observed in this system. Moreover, attachment to particles probably plays a strong role in controlling the transport of FIB in the system as well^35^. Particles in aquatic systems are often associated with nutrients and particulate carbon and phosphorus in particular, both of which can be limiting substrates for bacterial growth and in turn, can affect FIB survival in non-host environments^36^ and in systems with high particle loads, the TP pool is generally dominated by the particulate fraction, as is the case here. This, along with higher carbon concentrations in the particles probably confers a competitive advantage to the bacteria thereby enhancing their survival. However, for the moment, little other information exists on the proportions of attached and free FIB in other tropical water bodies and so it is difficult to compare our results with other tropical ecosystems. Nevertheless it also appears that TSS and TP plays an important role in determining the proportions of attached and free FIB in this highly turbid, tropical riverine system.

### Die-off rates

Schumacher^37^ in an in-stream incubation in the Upper Shoal Creek Basin, Southwestern Missouri, found that fecal coliform and EC densities decreased more than 90% from initial densities over a 42 h period. Troussellier *et al*.^38^ also reported rapid die-offs with over 90% of FIB lost over a 128 h period. We also observed a rapid decrease in EC and TC over the 5 day incubation, with in many cases up 100% loss after 120 h. We therefore chose to calculate the decay constants over the first 4 days. Our TC and EC die-off rate decay constants (k) ranged from a minimum of 0.01 d^−1^ to a maximum of 1.13 d^−1^ for EC and from 0.17 d^−1^ to 1.33 d^−1^ for TC, with a mean of 0.36 ± 0.21 d^−1^ for EC_free_ and 0.44 ± 0.23 d^−1^ for EC_tot_ and 0.83 ± 0.19 d^−1^ for TC_free_ and 0.61 ± 0.28 d^−1^ for TC_tot_. These rates are similar to other studies from temperate aquatic environments. For example, Menon *et al*.^39^, working in the Seine river, France observed die-off rates of 0.19 to 0.82 × 10^−3^ h^−1^ for EC and Blaustein *et al*.^40^, in a review on EC survival in a range of temperate aquatic environments reported an average rate of 0.725 ± 0.078 d^−1^. In one of the few articles dealing with die-off rates in sub-tropical systems, Chan *et al*.^41^ also found values of between 0.85 to 1.50 d^−1^ for the coastal water around Hong Kong, similar to the values we report in this work.

We observed higher die-off rates for TC_free_ than for TC_tot_, however, this was not the case for EC, where die-off was significantly higher for EC_tot_ than for EC_free_. Some authors^42^,^43^ have shown lower decay rates for fecal bacteria attached to sediments as compared to free fecal bacteria, as we show here. Attachment to particles may protect FIB against grazing by small heterotrophic nanoflagellates (the main grazers of bacteria in aquatic systems) as well as providing a micro-environment rich in nutrients. Although, according to Sinton *et al*.^44^ and Chan *et al*.^41^, exposure to solar radiation and predation are among the most important factors controlling die-off, although it seems from our work that attachment to particles can also be an important factor at least for TC.

We observed systematically higher die-off rates for TC_tot_ than for EC_tot_. Why this might be the case in this system merits some reflection. The appropriateness of using TC and EC as indicators of faecal contamination in tropical systems has been questioned by several authors e.g.^45^,^46^. Indeed, it has been shown that *E.coli* may be able to persist and even proliferate for some time in tropical freshwaters^47^,^48^,^49^ particularly in those with high temperatures and elevated nutrient and organic matter concentrations. This may well explain the differences in die-off rates between EC and TC. It may also be why die-off in EC_free_ was lower than that for EC_att_. Although the technique used in this work allowed us to determine the presence or absence of TC or EC, it provides no information on the sources of the TC or EC. Therefore, if the FIB are from different origins with different levels of adaptation to the environment, then this may explain some of the differences between the die-off rates of free and attached TC and EC. Nevertheless, this clearly is a hypothesis that merits further investigation *in situ* using some of the newer microbial source tracking techniques^36^.

Many studies have indicated that seasonal variations can influence FIB die-off rates through modifications of temperature, pH, nutrient concentration, and dissolved oxygen^50^,^51^. In this study, die-off rates were determined over the whole year with temperatures varying between 9.5 and 35 °C and we did not observe any significant relationship with *in situ* temperature. Moreover, we only observed a significant correlation between temperature and TC_free_ in contrast to what has been found by other investigators^23^,^52^. We did, however, find significant differences in FIB numbers and die-offs between the wet and dry seasons, emphasizing the dominant role of the hydrology. Why we did not find a clear temperature relationship for FIB number or die-off rate is not clear from the data, however, it is probably related to other environmental and source related factors not investigated in this work.

## Conclusions

There have been many studies on the survival of EC and other indicators of faecal pollution in freshwater, marine and estuarine habitats in temperate ecosystems however less information exists for tropical aquatic environments. Crane and Moore^53^ observed that the identification of relationships between environmental and physical parameters and FIB survival was a fundamental topic for future research in the field. Although their work is dated, and much work has been published since on temperate ecosystems, it is still the case that very little information is available on die-off rates in tropical and sub-tropical ecosystems where the management of FIB concentration is most needed and where the risks to human populations are highest^36^. In our work from the sub-tropical Red River system in Viet Nam, we found that FIB numbers exceed Vietnamese water quality guidelines of 20 and 150 colonies 100 ml^−1^ for EC and TC, respectively, throughout the whole year at almost all of the 10 stations investigated. Moreover, many values exceeded 500 colonies 100 ml^−1^ above which the World Health Organization considers that there is a 10% risk of gastro-intestinal illness after one single exposure. Therefore, the use of water from sites with high FIB numbers such as those in the downstream sites pose a real risk to public health. We also found a significant correlation between particles (as TSS or TP) and FIB as has been observed in temperate riverine ecosystems. However, in contrast to other studies we did not find a significant correlation between FIB and temperature for this subtropical environment, but instead a correlation with discharge (wet vs. dry season). Indeed, the highest TSS concentrations and FIB numbers were found during the wet season at high discharge. It is therefore probable that in developing countries where sanitation facilities are deficient and where people and their livestock live in close proximity to the river, FIB contamination mostly originates from diffuse sources^21^. Nevertheless, the data presented here, notably that of FIB numbers and their respective die-off rates, provides a base for the application of a model that will allow the parameterization of FIB dynamics in Red River and delta and potentially in other large river basins of the sub-tropical and tropical belt.

## Additional Information

**How to cite this article**: Nguyen, H. T. M. *et al*. Seasonal variability of faecal indicator bacteria numbers and die-off rates in the Red River basin, North Viet Nam. *Sci. Rep.* **6**, 21644; doi: 10.1038/srep21644 (2016).

## Supplementary Material

Supplementary Information

## Figures and Tables

**Table 1:** Location and characteristics of the surrounding areas at the 10 stations.

Station	Sampling location		River	Characteristics
	Latitude	Longitude		
Yen Bai	21°42'	104°53'	Thao river (upstream Red River)	Opposite a traditional brick factory that uses coal and mud and upstream of the agro-industrial processing zones of Van Yen (40 km) and the Tran Yen urban district (14 km). The sample was collected at 80 m from the bank. Water depth at this site is 5 m.
Vu Quang	21°34'	105°15'	Lo river	Low population area. The sample was collected at 130 m from the bank. Water depth is 17 m.
Hoa Binh	20°49'	105°19'	Da river	Site is 5.5 km upstream of the Hoa Binh hydroelectric dam. Sample was collected at 300 m from the bank. Water depth at this site is 10 m and the river banks are formed of weathered rock.
Son Tay	21°09'	105°52'	Red river	Site is 300 m upstream of the Son Tay coal ports. Sample was collected at 60 m from the bank, water depth 12 m.
Ha Noi	21°02'	105°51'	Red river	Site is 50 m downstream of the Chuong Duong bridge in Hanoi city. Sample was collected at 10m from the bank, water depth was 1m. This station is downstream of the confluence of the Da, Thao and Lo rivers.
Gian Khau	20°19'	105°55'	Day river	Site is 2 km from the Gian Khau industrial zone and Visai coal clinker port. Sample was collected at 38 m from the bank. This station is in a peri-urban area of the Red River delta.
Quyet Chien	20°30'	106°15'	Tra Ly river	Site is 2 km upstream from poultry and fish farms. Sample was collected at 35 m from the left bank, water depth was 7 m.
Nam Dinh	20°25'	106°10'	Dao river	Site is opposite a factory that produces construction materials. Sample was collected at 100 m from the left bank, water depth was 5 m.
Truc Phuong	20°19'	106°16'	Ninh Co river	Site is 7 km upstream of Nam Dinh city from which it receives sewage. Traditional silk spinning villages near the river release effluent containing silk chemicals and silkworm cocoon waste. Sample was collected at 100 m from the bank.
Ba Lat	19°30'	106°00'	Red river	Site is 7 km downstream of the Ba Lat seaport. Sample was collected at 300 m from the bank, water depth was 8 m. Site is under tidal influence.

River depth (m) at the sampling site is also provided. All samples were collected from the surface layer as grab samples.

**Table 2:** Average (±se) die-off rates for EC_tot_ and EC_free_ and TC_tot_ and TC_free_ (k, d^−1^) in the Red river basin.

Decay rate(d^–1^)	Yen Bai		Ha Noi		Gian Khau		Truc Phuong	
	ave	se	ave	se	ave	se	ave	se
k EC_tot_	0.59Bab	0.31	0.31Aa	0.13	0.36Aab	0.06	0.60Ba	0.21
k EC_free_	0.50BCa	0.28	0.25Aa	0.10	0.25ABa	0.09	0.54Ca	0.229
k TC_tot_	0.85Bb	0.21	0.57ABab	0.21	0.47Abc	0.18	0.55Aa	0.35
k TC_free_	0.77Aab	0.24	0.90Ab	0.13	0.79Ac	0.20	0.84Aa	0.21

The values were calculated for the first 4 days of the incubation. Different capital letters indicate a significant difference between station and different lowercase letters indicate a significant difference between decay rates of EC_tot_, EC_free_,TC_tot_ and TC_free_ within the same station.

**Table 3:** Pearson’s correlation matrix for the environmental variables and FIB.

Variables	EC_free_ log(col.100 ml^−1^)	EC_att_ log(col.100 ml^−1^)	EC_tot_ log(col.100 ml^−1^)	TC_free_ log(col.100 ml^−1^)	TC_att_ log(col.100 ml^−1^)	TC_tot_ log(col.100 ml^−1^)
pH	−0.112	−0.168	−0.170	−0.017	0.039	0.001
T (°C)	0.147	0.017	0.054	**0.196**	0.137	0.176
DO (mg l^−1^)	0.060	0.006	0.039	0.024	0.095	0.042
Cond (μs cm^−1^)	0.093	0.151	0.123	0.012	0.108	0.038
TSS (mg l^−1^)	**0.238**	**0.224**	**0.216**	**0.247**	**0.236**	**0.293**
NH_4_ (mg l^−1^)	−0.008	0.026	−0.016	0.100	−0.090	0.098
PO_4_ (mg l^−1^)	−0.081	**−0.260**	−0.169	**−0.314**	−0.011	**−0.251**
TP (mg l^−1^)	**0.252**	**0.230**	**0.222**	**0.270**	**0.262**	**0.322**

The correlation was performed with the log(TC or EC) values.

Values in bold indicate significance at p < 0.05.

**Table 4:** Pearson’s correlation matrix for the environmental variables and k(d^−1^) for the free and total (attached + free) TC and EC.

Variables	EC_tot_ die-off k (d^−1^)	EC_free_ die-off k (d^−1^)	TC_tot_ die-off k (d^−1^)	TC_free_ die-off k (d^−1^)
pH	0.078	0.078	0.043	0.096
T (°C)	0.079	0.070	0.046	0.256
DO (mg l^−1^)	0.181	0.160	0.014	0.082
Cond (μs cm^−1^)	−0.060	−0.213	−0.158	−0.086
TSS (mg l^−1^)	**0.405**	0.213	0.195	0.025
NH_4_ (mg l^−1^)	−0.024	−0.072	0.208	−0.208
PO_4_ (mg l^−1^)	−0.130	−0.123	−0.112	0.065
TP (mg l^−1^)	**0.424**	0.223	**0.272**	−0.059

The k(d^−1^) was determined over 4 days.

Values in bold indicate significance at p < 0.05.
