# Supplementary material for: Seasonal variability of faecal indicator bacteria numbers and die-off rates in the Red River basin, North Viet Nam
Source: Sci Rep. 2016 Feb 12;6:21644. doi: 10.1038/srep21644 (PMC4751496; doi:10.1038/srep21644)
Supplement: Supplementary Information [file srep21644-s1.pdf]

Supplementary materials

Title: Seasonal variability of faecal indicator bacteria numbers and die-off rates in the Red River basin, North Viet Nam

Authors: Huong Thi Mai Nguyen, Quynh Thi Phuong Le, J. Garnier, J.-L. Janeau, E. Rochelle-Newall.

Table S1. The ranges (min-max; x 10^2 colonies 100 ml^{-1}) of free, attached and total EC and TC (EC_{free}, EC_{att} and EC_{tot}, and TC_{free}, TC_{att} and TC_{tot} , respectively) and the percentage of attached EC and TC (%EC_{att} and %TC_{att}, respectively) at the ten stations for the study period.

Station	EC_{free}	EC_{att}	EC_{tot}	%EC_{att}	TC_{free}	TC_{att}	TC_{tot}	%TC_{att}
	x 10^2 colonies 100 ml^{-1}			(%)	x 10^2 colonies 100 ml^{-1}			(%)
Yen Bai	0.5 – 103.5	0.5 – 49	1.5-152.5	23.1 – 77.8	0 – 206.5	1 – 130.5	37.5-391	7.0 – 60.9
Hoa Binh	0 – 7	1 – 3	0-2	50 – 100	0 – 15	1 – 9	5-17	14.3 – 52.9
Vu Quang	0 – 5	1 – 4	2-7	28.6 – 66.7	1 – 128	3 – 20	5-148	7.7 – 80
Son Tay	0.5 – 10	1 – 7	1-10	20 – 75	1 – 123	3 – 28	6-55	16.7 – 80
Ha Noi	0.5 – 7.5	0.5 – 4.5	1-11	15.4 – 80	6.5 – 31	3.5 – 31	10-57.5	32.4 – 60.1
Gian Khau	0.5 – 4.5	1 – 2.5	4-6	18.2 – 55.6	5 – 31.5	0.5 – 8.5	7.5-29.0	6.7 – 60.7
Quyet Chien	0 – 5	1 – 3	0-5	33.3 – 66.7	0 – 219	0 – 8	3-225	2.7 – 66.7
Nam Dinh	0 – 70	2 – 10	7-31	9.7 – 50	2 – 161	2 – 37	6-62	2.7 – 69.2
Truc Phuong	0 – 34	0.5 – 16.5	5.5-48	10.5 – 54.5	4 – 146	3 – 32	10-150.5	3 – 54.2
Ba Lat	0 – 40	1 – 19	10-50	20 – 87.5	4 – 81	2 – 71	8-71	10 – 53.3
